# Cucurbitacin E Exerts Anti-Proliferative Activity via Promoting p62-Dependent Apoptosis in Human Non-Small-Cell Lung Cancer A549 Cells

**DOI:** 10.3390/cimb45100514

**Published:** 2023-10-07

**Authors:** Han-Lin Hsu, Bo-Jyun Lin, Yu-Chen Lin, Chih-Chieh Tu, Nham-Linh Nguyen, Ching-Chiung Wang, Mei-Chuan Chen, Chun-Han Chen

**Affiliations:** 1 Division of Pulmonary Medicine, Department of Internal Medicine, Wan Fang Hospital, Taipei Medical University, Taipei 116, Taiwan; 2 Pulmonary Research Center, Wan Fang Hospital, Taipei Medical University, Taipei 116, Taiwan; 3 School of Respiratory Therapy, College of Medicine, Taipei Medical University, Taipei 110, Taiwan; 4 Department of Pharmacology, School of Medicine, College of Medicine, Taipei Medical University, Taipei 110, Taiwan; 5 Graduate Institute of Medical Sciences, College of Medicine, Taipei Medical University, Taipei 110, Taiwan; 6 Faculty of Chemical and Food Technology, HCMC University of Technology and Education, Ho Chi Minh 70000, Vietnam; 7 School of Pharmacy, College of Pharmacy, Taipei Medical University, Taipei 110, Taiwan; 8 Traditional Herbal Medicine Research, Center of Taipei Medical University Hospital, Taipei 110, Taiwan; 9 Ph.D. Program in Clinical Drug Development of Herbal Medicine, College of Pharmacy, Taipei Medical University, Taipei 110, Taiwan; 10 Graduate Institute of Pharmacognosy, Taipei Medical University, Taipei 110, Taiwan; 11 Cell Physiology and Molecular Image Research Center, Wan Fang Hospital, Taipei Medical University, Taipei 116, Taiwan

**Keywords:** Cucurbitacin E, NSCLC, autophagy, apoptosis, ROS

## Abstract

EGFR tyrosine kinase inhibitors (TKIs) are the first-line treatment for advanced EGFR-mutated non-small-cell lung cancer (NSCLC). However, NSCLC patients with wild-type EGFR and KRAS mutation are ineligible for EGFR-TKIs. Therefore, the discovery of new therapeutic agents is urgently needed for NSCLC patients who cannot receive targeted therapies. Natural products possess tremendous chemical diversity and have been extensively investigated for their anticancer activity. In this study, we found that Cucurbitacin E (Cu E), a triterpene of cucurbitacins widely presented in the edible plants of the Cucurbitaceae family, significantly inhibits the viability and proliferation of A549 cells that harbor wild-type EGFR and KRAS mutation. Our results revealed that Cu E increases cell-cycle arrest at G2/M and subG1 phase. Mechanistically, Cu E significantly inhibits the phosphorylation and protein levels of regulatory proteins and hinders G2/M cell-cycle progression. Meanwhile, the treatment of Cu E resulted in DNA damage response and apoptosis. For the first time, we observed that Cu E induces incomplete autophagy as evidenced by increased LC3B-II expression and p62-accumulation. Knockdown of p62 rescued the cells from Cu E-mediated anti-proliferative effect, apoptosis, DNA damage, and ROS production. These findings suggest that Cu E is a promising drug candidate for NSCLC.

## 1. Introduction

Lung cancer is the second most commonly diagnosed cancer and the leading cause of cancer death worldwide in both men and women [1]. In 2023, it is estimated that 238,340 people will be diagnosed, and 127,070 people will die from the disease in the USA [2]. Therefore, there is still room for improvement in the diagnosis and treatment of lung cancer. According to the histological type, about 80–85% of cases are non-small-cell lung carcinoma (NSCLC). NSCLC can be further divided into lung adenocarcinoma, squamous-cell carcinoma, and large-cell carcinoma according to the histological characteristics. The other 15% of patients have small-cell lung cancer, which is more aggressive with a worse prognosis [3]. Common treatments for lung cancer include palliative care, surgery, chemotherapy, and targeted therapy. Recent advancements in molecular and genetic technology have allowed for further guidance in lung cancer treatment based on genetic mutations and molecular characteristics. Frequent genetic abnormalities in NSCLC involve EGFR, PI3K/AKT/mTOR, RAS/MAPK, and NTRK/ROS1 pathways [4]. The targeted therapies currently approved for NSCLC treatment include EGFR, ALK, ROS1, BRAF, RET, NTRK, and HER2 inhibitors [5]. Activating EGFR mutations, such as exon 19 deletions and exon 21 L858R point mutations, are the most common oncogenic alterations, with a much higher prevalence in Asian NSCLC populations compared to Western populations. EGFR tyrosine kinase inhibitors (TKIs) are the first-line treatment options for advanced EGFR-mutated NSCLC. However, patients inevitably encounter acquired resistance to targeted therapy due to on-target mutations, such as EGFR T790M (with first-or second-generation EGFR-TKIs) and EGFR C797S (with third-generation EGFR-TKIs), or activation of bypass signaling pathway such as MET amplification [6,7]. Furthermore, NSCLC patients with wild-type EGFR are not eligible for EGFR-TKIs, and certain gene alternations such as KRAS mutations, in-frame insertion mutations in exon 20 of HER2, and loss of PTEN, can cause primary resistance [8]. Therefore, the development of new therapeutic approaches is urgently needed for NSCLC patients who cannot receive targeted therapies.

Natural products and their derivatives are discovered in plants, animals, and microorganisms, exhibiting tremendous chemical diversity. Consequently, they have been extensively studied for their anticancer activity for over half a century [9]. Statistically, 32% of all small-molecule drugs approved between 1981 and 2019 were natural products or their derivatives [10]. The plant kingdom is particularly significant as a source of biologically active compounds, with approximately 1000 different plant species demonstrating anticancer properties [11]. Chemotherapeutic drugs derived from natural compounds, such as paclitaxel, doxorubicin, and camptothecin, have been successfully utilized in the treatment of lung cancer patients [12]. Meanwhile, the process of carcinogenesis involves multiple factors, including genetic and epigenetic changes, as well as developmental stages [13,14]. Consequently, multi-target drugs, rather than highly selective compounds, offer opportunities for controlling multifactorial malignancies and achieving clinical efficacy [15,16]. Natural products have proven effective in targeting numerous signaling pathways that contribute to the survival of malignant cells [17]. Some drugs derived from natural products have been investigated to address unmet medical needs. For instance, ixabepilone, a semisynthetic analog of epothilone B, has been approved for advanced or metastatic breast cancer after the failure of anthracycline and taxane treatments, either in the adjuvant or metastatic setting [18]. Eribulin, a completely synthetic halichondrin B derivative, has demonstrated significant activity and efficacy in heavily pretreated refractory breast cancer patients [19]. These findings underscore the continued importance of natural products as attractive sources for the discovery of anticancer drugs.

Cucurbitacins belong to a large family of tetracyclic triterpenoids and are widely presented in the edible plants of the Cucurbitaceae family, including cucumber, melon, watermelon, and pumpkin. Several pharmacological activities of cucurbitacins have been reported, including those that are lipid lowering, hypolipidemic, antidiabetic, anti-inflammatory, hepatic protection, and antitumor [20]. Based on the chemical structure, cucurbitacins are classified into 12 groups, starting with the letter A to T [21]. Among these, cucurbitacin B, D, E, I, IIa, L glucoside, Q, and R exhibit the most significant anticancer activity [22]. Cucurbitacin E (Cu E) is a highly oxygenated triterpene of cucurbitacins, harboring a variety of pharmacological effects such as hepatoprotective, anti-inflammatory, neuroprotective, and anti-cancer activities [23,24,25,26]. Recent studies revealed that Cu E inhibits proliferation and metastasis of cancer cells by causing cell-cycle arrest and apoptosis, as well as suppressing PI3K/Akt/mTOR, JAK/STAT3, and MAPK pathways [27,28,29]. Interestingly, Cu E reportedly induces non-apoptotic cell death, such as senescence and autophagy in cancer cells [30,31]. Cu E has also been found to exert anti-angiogenic effects via the blockage of VEGFR2-mediated JAK2–STAT3 signaling and prevention of actin polymerization [32,33]. However, the direct intracellular potential targets of Cu E remain unknown, and it is important to further elucidate the molecular mechanisms underlying Cu E-mediated anticancer effects to optimize its potential therapeutic applications.

Autophagy is an evolutionarily conserved cellular process that plays a critical role in maintaining cellular homeostasis under both physiological and pathological conditions [34]. This process involves the sequestration of cellular components into double-membraned vesicles called autophagosomes, which are then transported to lysosomes for degradation and recycling [35]. By removing damaged organelles, protein aggregates, and invasive pathogens, autophagy preserves cellular integrity and facilitates adaptation to stress. However, dysregulation of autophagy has been implicated in the development of various diseases, including cancer, neurodegenerative disorders, inflammation, and metabolic syndromes [36]. As a result, autophagy has become an attractive target for the development of novel therapeutic strategies against these conditions [37]. Several studies have investigated the effects of Cu E on autophagy, although its impact on cell death remains controversial. Some findings suggest that Cu E inhibits the PI3K/AKT/mTOR signaling pathway, leading to the activation of autophagy in human cancer cells and intestinal epithelial cells [31,38]. Conversely, Cu E has been found to induce protective autophagy mediated by ROS in lung 95D cells [39]. Additionally, the combination of Cu E and myricetin, an active component of *C. colocynthis*, exhibits a synergistic effect and suppresses autophagy in NSCLC cells [40]. While these findings suggest a potential relationship between Cu E and autophagy, further studies are necessary to fully understand the underlying mechanisms and establish the therapeutic implications of Cu E-induced autophagy in NSCLC. In this study, we have discovered that Cu E promotes incomplete autophagy, which is crucial for subsequent ROS production, DNA damage, and apoptosis. Overall, our findings demonstrate the potential of Cu E as a promising drug candidate for NSCLC.

## 2. Materials and Methods

### 2.1. Cell Culture and Reagents

A549 (EGFR-WT, KRAS mutation), NCI-H1299 (EGFR-WT, KRAS-WT), and NCI-H226 (EGFR-WT, KRAS-WT) cells were purchased from the American Type Culture Collection (ATCC) (Manassas, VA, USA). NCI-H1437 (EGFR-WT, KRAS-WT) and BEAS-2B (normal human bronchial epithelial cell line) cells are kind gifts from Professor Ming-Jen Hsu at the Department of Pharmacology at Taipei Medical University. A549, NCI-H1299, NCI-226, and NCI-H1437 cells were maintained in 10% fetal bovine serum (FBS)-supplemented RPMI 1640 medium and 1 × antibiotic–antimycotic (Thermo Fisher Scientific, Waltham, MA, USA), and BEAS-2B cells were maintained in bronchial epithelial growth medium (Lonza, Walkersville, MD, USA) at 37 °C in a humidified incubator containing 5% CO_2_. Cucurbitacin E was purchased from MedChem Express (Monmouth Junction, NJ, USA).

### 2.2. Cell Viability and Sulforhodamine B (SRB) Assay

The cells were seeded at 5000 cells per well on 96-well plates and allowed to adhere overnight. Subsequently, they were treated with DMSO (0.05%) or the indicated compounds without changing the media for 48h. Cell viability was assessed using the MTT (3-(4,5-Dimethylthiazol-2-yl)-2,5-diphenyltetrazolium bromide) assay, as previously described [41]. The absorbance of the treatment group was compared to that of the DMSO-treated control group, which was considered 100%, to calculate cell viability. The IC_50_ value represents the drug concentration that resulted in a 50% reduction in cell viability. Cell proliferation was measured using the SRB assay, following a previously described protocol [42]. The growth inhibition of 50% (GI_50_) was determined as the drug concentration that led to a 50% reduction in the total protein increase compared to control cells during the incubation with the compound.

### 2.3. Lentivirus System

Lentiviral particles containing plasmids of shRNA against ATG5 (TRCN0000151474, TRCN0000151963) and p62 (#1, TRCN0000007236; #2, TRCN0000007237) were obtained from the National RNAi Core Facility (Academia Sinica, Taipei, Taiwan). A549 cells were seeded into a 6-well plate for transduction. Stable knockdown cells were selected and maintained with 2 μg/mL puromycin (InvivoGen, San Diego, CA, USA).

### 2.4. Flow Cytometry

For cell-cycle analysis, cells were seeded at 1 × 10^5^~2 × 10^5^ cells per well on 6-well plates and incubated overnight. The following day, cells were treated with DMSO (0.05%) or various concentrations of compounds for the indicated time without changing the media. After the treatment, cells were harvested by trypsinization, washed with 1 mL of phosphate-buffered saline, and fixed in ice-cold 75% ethanol at −20 °C overnight. Subsequently, the cells were stained with propidium iodide (80 µg/mL) containing 0.1% Triton-X 100 and 100 µg/mL of RNaseA. DNA content was analyzed using FACScan and CellQuest software (Becton Dickinson, Mountain View, CA, USA). For ROS analysis, cells were stained with 0.1 μmol/L H_2_DCFDA (Biotium, Fremont, CA, USA) at 37 °C for 20 min. After washing with PBS three times, the cells were subjected to ROS detection via FACScan and CellQuest Pro 4.0.2 software.

### 2.5. Western Blot

Cells were seeded at 1 × 10^5^~2 × 10^5^ cells per well on 6-well plates overnight and treated with DMSO (0.05%) or indicated compound at various concentrations of Cu E for the indicated time without changing the media. Following treatment, proteins were extracted from whole-cell lysates using a lysis buffer. Equal amounts of protein were separated by SDS-PAGE and immunoblotted using specific antibodies, as previously described [42]. The complete blots can be found in the Supplementary Data. Antibodies against various proteins were obtained from the following sources: antibodies against various proteins were obtained from the following sources: Novus (Littleton, CO, USA) for caspase 3, LC3, and GAPDH; Cell Signaling Technology (Danvers, MA, USA) for p-Aurora A/B/C, p-cdc2(Y15), p-cdc2(T161), p-cdc25c(S216), PARP, γH2AX, and ATG5; Santa Cruz (Dallas, TX, USA) for cdc2, cyclin B1, and PLK1; Millipore (Billerica, MA, USA) for p-MPM2. The band intensities of each protein were determined by using the Image J software (Version 1.51, National Institutes of Health, Bethesda, MD, USA), and normalized with the intensity of loading control.

### 2.6. Statistics and Data Analysis

Each experiment was performed at least two times, and presentative data are shown. Data in the bar graph are given as the means ± S.D. Means were checked for statistical difference using the Student’s *t*-test and *p*-values less than 0.05 were considered significant (* *p* < 0.05, ** *p* < 0.01, *** *p* < 0.001).

## 3. Results

### 3.1. The Effects of Cu E on the Viability and Proliferation in A549 Cells

**Figure 1:**
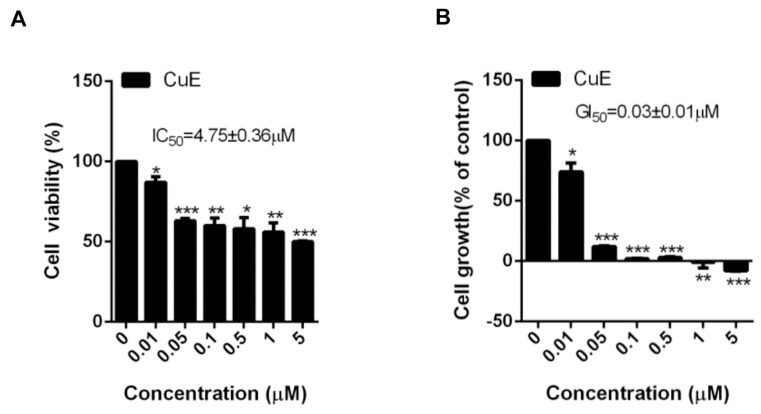
Effects of Cu E on cell viability and proliferation in A549 cells. The cells were treated with different concentrations of Cu E for 48 h. (**A**) Cell viability was determined through MTT assay. (**B**) Cell growth was determined through SRB assay. The data are presented as the mean ± S.D. (*n* = 2) * *p* < 0.05, ** *p* < 0.01, and *** *p* < 0.001 compared with control group.

Firstly, we employed A549 cells as a model for EGFR-TKI-resistant lung adenocarcinoma, which harbors wild-type EGFR and KRAS mutation [43]. To investigate the anticancer activity of Cu E, we evaluated cell viability and proliferation using the MTT and SRB assays, respectively. The data clearly demonstrated that Cu E exerted a significant suppressive effect on the viability of A549 cells (Figure 1A, IC_50_ = 4.75 ± 0.36 μM) and also inhibited their proliferation (Figure 1B, GI_50_ = 0.03 ± 0.01 μM) after a 48-h treatment. Meanwhile, Cu E also exhibits anti-proliferative effects in other NSCLC cells expressing wild-type EGFR, such as NCI-H1299, NCI-H226, and NCI-H1437 cells (Figure S1). Importantly, Cu E showed less anti-proliferative activity in the normal human bronchial epithelial BEAS-2B cells with a GI_50_ value of 0.85 ± 0.03 μM (Figure S1).

### 3.2. Cu E Increased Cell-Cycle Arrest at the G2/M and subG1 Phase in A549 Cells

**Figure 2:**
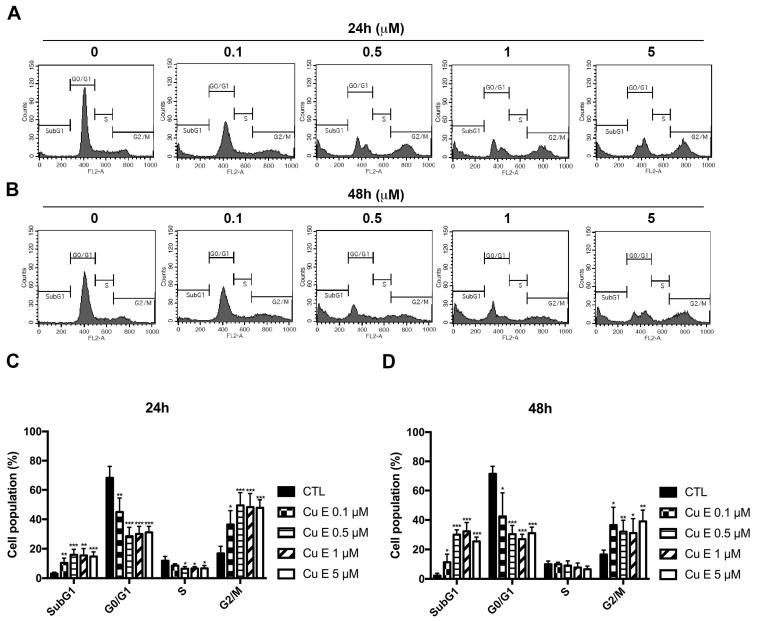
Cu E increases cell-cycle arrest at G2/M and subG1 phase in A549 cells. The cells were treated with different concentrations of Cu E for 24 h (**A**,**C**) and 48 h (**B**,**D**). Cell-cycle distribution was analyzed using flow cytometry after propidium iodide (PI) staining. The data are presented as the mean ± S.D. (*n* = 4) * *p* < 0.05, ** *p* < 0.01, and *** *p* < 0.001 compared with control group.

To further investigate the effect of Cu E on the cell cycle distribution in A549 cells, we performed PI staining and flow cytometry analysis. The data revealed that Cu E increased the populations of cells in the subG1 and G2/M phases in a concentration-dependent manner after 24 h (Figure 2A,C) and 48 h (Figure 2B,D) of treatment. Additionally, a time-course study demonstrated that Cu E induced cell-cycle arrest at the G2/M phase as early as 12 h (Figure S2A,B), and the accumulation of cells in the subG1 phase reached its maximum effect at 48 h (Figure S2A,C). Overall, these findings indicate that Cu E promotes cell-cycle arrest at the G2/M phase and increases the number of cells in the subG1 phase in A549 cells.

### 3.3. The Effects of Cu E on the Levels of Cell-Cycle Regulatory Proteins in A549 Cells

**Figure 3:**
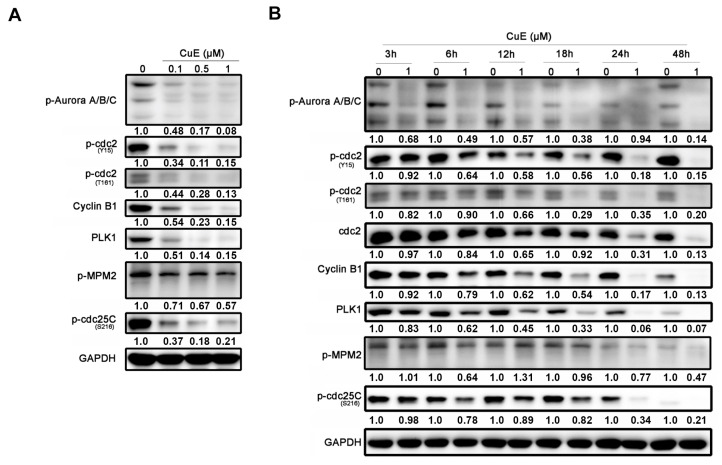
The effects of Cu E on the levels of cell-cycle regulatory proteins in A549 cells. (**A**) A549 cells were treated with indicated concentrations of Cu E for 24 h, and cell lysates were subjected to Western blotting using indicated antibodies. (**B**) A549 cells were exposed to the 1 μM of Cu E for indicated times, and cell lysates were subjected to Western blotting using indicated antibodies. Equal loading of protein was confirmed by GAPDH expression. Band intensities of each protein were determined by Image J, and normalized over GAPDH. Fold changes compared to control group were depicted.

Based on the previously mentioned data, it was observed that Cu E increased the cell population in the G2/M phase in A549 cells. The G2/M phase transition is regulated by various cell-cycle regulatory proteins. CDK1, also known as cdc2, forms a complex with cyclin B, playing a crucial role in driving the G2/M phase transition. Wee1 kinase phosphorylates and inhibits CDK1, while cdc25 removes inhibitory phosphorylation, leading to CDK1 activation [44]. Aurora kinases and PLKs are also involved in the intricate regulation and functional interplay during the G2/M transition [45]. To further investigate the effects of Cu E on cell-cycle regulatory proteins, Western blotting was performed. The results showed that Cu E decreased the phosphorylation of both the activating site (T161) and inhibitory site (Y15) of cdc2, as well as the expression of cyclin B1 in a concentration-dependent manner. Moreover, Cu E suppressed the phosphorylation of aurora A/B/C, mitotic protein (MPM2), and cdc25c (S216), along with the protein level of PLK1 (Figure 3A). Notably, the phosphorylation of aurora A/B/C was inhibited by Cu E within 3 h. The phosphorylation of cdc2 (T161, Y15), protein levels of cdc2, cyclin B1, and PLK1 were reduced after 12 h of treatment (Figure 3B). The phosphorylation of MPM2 and cdc25c (S216) was also suppressed by Cu E after 24 h of treatment (Figure 3B). Overall, these findings suggest that Cu E significantly inhibits the phosphorylation and protein levels of cell-cycle regulatory proteins, thus impeding G2/M cell-cycle progression.

### 3.4. The Effects of Cu E on Apoptosis in A549 Cells

**Figure 4:**
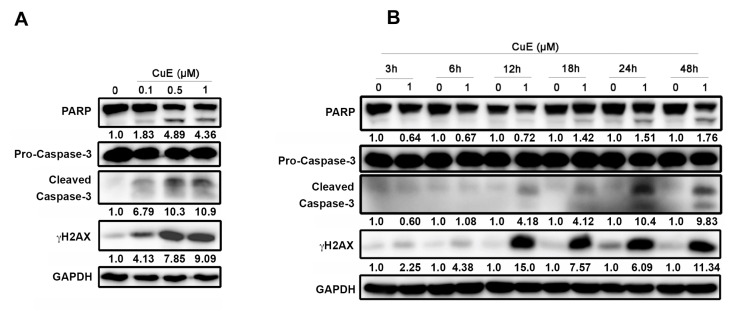
The effects of Cu E on apoptosis in A549 cells. (**A**) A549 cells were treated with indicated concentrations of Cu E for 48 h, and cell lysates were subjected to Western blotting using indicated antibodies. (**B**) A549 cells were exposed to the 1 μM of Cu E for indicated times, and cell lysates were subjected to Western blotting using indicated antibodies. Equal loading of protein was confirmed by GAPDH expression. Band intensities of each protein were determined by Image J, and normalized over GAPDH. Fold changes compared to control group were depicted.

Based on the aforementioned observations, it was observed that Cu E increased the population of cells in the subG1 phase (Figure 2), indicating the induction of apoptosis. Previous studies have shown that Cu E inhibits the proliferation and metastasis of cancer cells by inducing cell-cycle arrest and apoptosis [27,28,29]. Therefore, we further investigated the effects of Cu E on apoptosis using Western blotting. As expected, Cu E increased the activation of caspase-3, PARP, and the DNA double-strand break marker γH2AX in a concentration-dependent manner (Figure 4A). Time-course analysis revealed that Cu E promoted the induction of γH2AX within 12 h, while apoptotic markers were induced between 18 and 48 h of treatment (Figure 4B). Collectively, these findings suggest that the treatment of Cu E leads to DNA damage response and apoptosis in A549 cells.

### 3.5. The Effects of Cu E on Autophagy in A549 Cells

**Figure 5:**
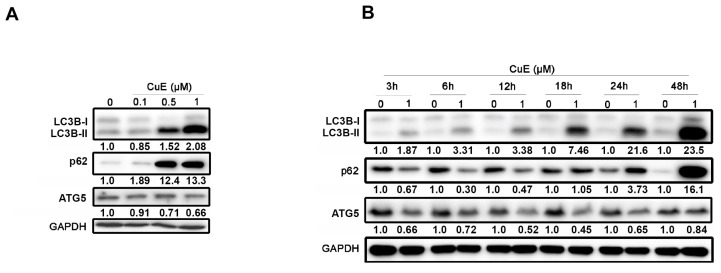
The effects of Cu E on autophagy in A549 cells. (**A**) A549 cells were treated with indicated concentrations of Cu E for 48 h, and cell lysates were subjected to Western blotting using indicated antibodies. (**B**) A549 cells were exposed to the 1 μM of Cu E for indicated times, and cell lysates were subjected to Western blotting using indicated antibodies. Equal loading of protein was confirmed by GAPDH expression. Band intensities of each protein were determined by Image J, and normalized over GAPDH. Fold changes compared to control group were depicted.

In addition to inducing apoptotic cell death, recent studies have demonstrated that Cu E can also promote autophagy by modulating key signaling pathways, such as the PI3K/Akt pathway and the mTOR signaling pathway [31,38]. To further investigate the effects of Cu E on autophagy in A549 cells, we examined the levels of regulatory proteins by Western blotting. The results showed that Cu E increased the expression of LC3B-II in a concentration-dependent manner after 48 h of treatment, indicating autophagy induction. Additionally, the cargo receptor p62 was also upregulated by Cu E, whereas the changes in the essential protein of autophagy, ATG5, were not appreciable (Figure 5A). Time-course analysis revealed that Cu E increased LC3B-II levels as early as 3 h of treatment, reaching its maximum effect at 48 h. Interestingly, the level of p62 decreased before 12 h of treatment but accumulated from 18 to 48 h of treatment (Figure 5B). These findings suggest that Cu E promotes autophagy within the first 12 h of treatment, but the process becomes incomplete from 18 to 48 h of treatment.

### 3.6. Cu E-Induced Incomplete Autophagy Is Crucial to ROS Production, DNA Damage, and Apoptosis in A549 Cells

**Figure 6:**
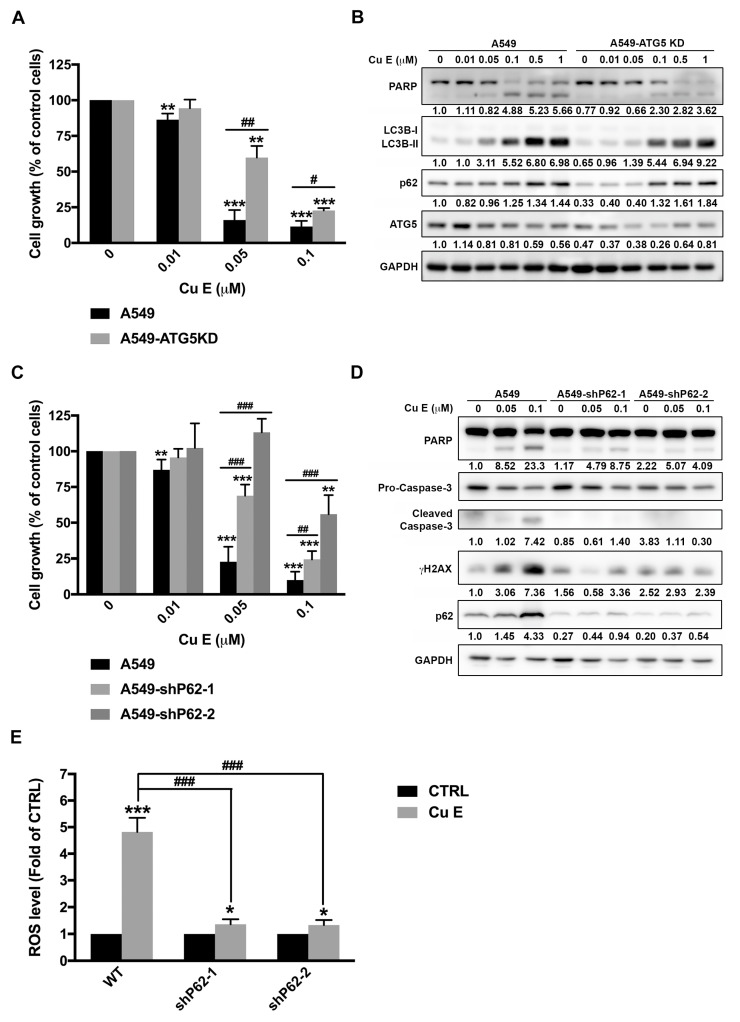
Cu E induces incomplete autophagy and ROS production in A549 cells. (**A**) A549 and A549-ATG5-KD cells were exposed to indicated concentrations of Cu E for 48 h, and cell growth was examined by SRB assay. The data are expressed as means ± S.D. (*n* = 4) **, *p* < 0.01; ***, *p* < 0.001 compared to control group; **^#^**, *p* < 0.05; **^##^**, *p* < 0.01 compared to A549 cells. (**B**) A549 and A549-ATG5-KD cells were exposed to indicated concentrations of Cu E for 48 h, and cell lysates were immunoblotted using the indicated antibodies. Band intensities of each protein were determined by Image J, and normalized over GAPDH. Fold changes compared to control group of wild-type A549 cells were depicted. (**C**) A549, A549-shP62-1, and A549-shP62-2 cells were exposed to indicated concentrations of Cu E for 48 h, and cell growth was examined by SRB assay. The data are expressed as means ± S.D. (*n* = 3~6) **, *p* < 0.01; ***, *p* < 0.001 compared to control group; **^##^**, *p* < 0.01; **^###^**, *p* < 0.001 compared to A549 cells. (**D**) A549, A549-shP62-1, and A549-shP62-2 cells were exposed to indicated concentrations of Cu E for 24 h, and cell lysates were immunoblotted using the indicated antibodies. Band intensities of each protein were determined by Image J, and normalized over GAPDH. Fold changes compared to control group of wild-type A549 cells were depicted. (**E**) Wild-type A549 (WT), A549-shP62-1, and A549-shP62-2 cells were exposed to 0.1 μM of Cu E for 24 h. Accumulation of intracellular ROS levels was stained with 0.1 μmol/L H_2_DCFDA and measured by flow cytometry. The data are presented as mean ± S.D. (*n* = 3) (*, *p* < 0.05; ***, *p* < 0.001 compared to control group; **^###^**, *p* < 0.001 compared to wild-type A549 cells).

Previous studies have demonstrated that Cu E induces cytoprotective autophagy [31,39]. To confirm the role of autophagy in the anti-cancer activity of Cu E, we used lentiviral transfection of shRNA to knock down the ATG5 gene, which is a key protein involved in isolation membrane expansion and essential for autophagosome formation [46] in A549 cells. Interestingly, ATG5 knockdown rescued Cu E-mediated anti-proliferative effect (Figure 6A), indicating that Cu E triggers pro-death autophagy. Although the knockdown efficiency of ATG5 is only 50%, we still observed that autophagy was partially abrogated in ATG5-knockdown cells under the treatment of Cu E at 0.05 mM and 0.1 mM, as evidenced by decreased levels of LC3B-II and p62 (Figure 6B). Moreover, Cu E-induced apoptosis was rescued by ATG5 knockdown (Figure 6B). To confirm the findings in ATG5-knockdown A549 cells, we further compared the effect of Cu E in wild-type (WT) and ATG5-knockout (ATG5-KO) mouse embryonic fibroblast (MEF) [47]. The data showed that ATG5-knockout rescued the cells from Cu E-mediated anti-proliferative activity (Figure S3A). We observed that Cu E triggered incomplete autophagy and apoptosis in WT-MEF, as shown by increased levels of LC3B-II, p62, and PARP cleavage, but this phenomenon was totally abrogated in ATG5-KO MEF (Figure S3B). These data further support our hypothesis that Cu E-induced incomplete autophagy contributes to apoptosis in A549 cells. Considering that Cu E promotes incomplete autophagy after 18 h of treatment (Figure 5A), we further knocked down the protein level of p62 in A549 cells to investigate the effect of incomplete autophagy on cell death. The data showed that p62 knockdown rescued A549 cells from Cu E-mediated anti-proliferative effect (Figure 6C) and apoptosis (Figure 6D). Furthermore, p62 knockdown reversed the Cu E-mediated increase in the DNA double-strand damage marker, γH2AX (Figure 6D). It has been reported that excessive ROS upregulates p62 levels and impairs cell survival [48,49]. Our previous study also revealed that incomplete autophagy triggers cell death through ROS elevation and DNA damage [50]. To investigate whether p62 accumulation is related to ROS production, which ultimately leads to Cu E-induced cell death, we examined the H_2_DCFDA-positive population. The data showed that Cu E significantly increased the H_2_DCFDA-positive population after 24 h of treatment, and this effect was reversed in p62-knockdown A549 cells (Figure 6E). These findings suggest that Cu E-induced incomplete autophagy is crucial for ROS production, DNA damage, and cell apoptosis.

## 4. Discussion

Lung cancer is a significant global health concern, being the second most commonly diagnosed cancer and the leading cause of cancer-related deaths [1]. While EGFR tyrosine kinase inhibitors (TKIs) are effective as first-line treatments for advanced EGFR-mutated non-small-cell lung cancer (NSCLC), the development of acquired resistance limits their long-term efficacy [6,7]. Furthermore, patients with wild-type EGFR or KRAS-mutated tumors are ineligible for EGFR-TKIs, creating an unmet need for alternative therapeutic options [8]. Natural products, known for their diverse chemical composition, have been extensively studied for their anticancer properties [9]. In this study, we focused on Cu E, a triterpene belonging to the cucurbitacins family. Our investigation revealed that Cu E significantly inhibits the viability and proliferation of A549 cells, which possess wild-type EGFR and a KRAS mutation [43]. It is worthwhile to investigate the anticancer activity of Cu E in colorectal cancer and pancreatic ductal adenocarcinoma, given that KRAS mutations are prevalent in these malignancies [51]. In this study, we have observed that Cu E inhibits the proliferation of NSCLC cells (NCI-H1299, NCI-H226, and H1437) expressing wild-type EGFR and wild-type KRAS (Figure S1). This observation suggests that pathways other than mutant KRAS may also play a role in its anti-cancer effects. Mechanistically, Cu E hinders G2/M cell-cycle progression by suppressing the phosphorylation and protein levels of key regulatory proteins. Additionally, our findings suggest that Cu E-induced incomplete autophagy plays a crucial role in promoting ROS production, DNA damage, and apoptosis. Based on these observations, Cu E emerges as a promising drug candidate for NSCLC.

In the current study, we made an interesting observation that Cu E induces cell-cycle arrest at the G2/M phase as early as 12 h of treatment (Figure S2A,B). This finding is consistent with the effects of Cu E on cell-cycle regulatory proteins, where we observed a significant decrease in the phosphorylation of aurora A/B/C, cdc2, and the protein levels of cdc2, cyclin B1, and PLK1 after 12 h of treatment (Figure 3B). These results suggest that Cu E effectively inhibits the phosphorylation and protein levels of regulatory proteins, thereby hindering G2/M cell-cycle progression. Importantly, our findings are in line with other studies that have demonstrated Cu E-induced G2/M cell-cycle arrest in various cancer types, including triple-negative breast cancer, glioblastoma, and bladder cancer [27,52,53]. However, the precise mechanism underlying how Cu E promotes cell-cycle arrest remains elusive. Previous studies have suggested that in brain cancer cells, cell-cycle arrest was achieved by downregulating the expression of cdc2 and cyclin B1 proteins and disrupting the cdc2/cyclin B1 complex through the upregulation of GADD45β [26]. Additionally, Cu E has been reported to inhibit tubulin polymerization, disrupt mitotic spindles, and induce cell-cycle arrest at the G2/M phase in Hela cells [54]. We also observed that Cu E induced hypodiploid aneuploidy, as evidenced by the presence of double peaks in the G0/G1 phase (Figure 2A,B). This phenomenon is consistent with mitotic spindle abnormalities [55]. Therefore, it would be worthwhile to investigate whether Cu E acts as an antimitotic agent, contributing to increased G2/M arrest and cell death in NSCLC.

Incomplete autophagy is defined as an impaired self-eating process in which accumulated autophagosomes are not able to fuse with lysosomes resulting in the blockage of autophagic flux [56]. Therefore, incomplete autophagy can lead to the accumulation of autophagosomes or defective cargo degradation, and promote cell death [46]. Targeting incomplete autophagy by small molecule drugs may contribute to the development of novel generation therapeutic agents for cancer patients [56]. In this study, we observed that Cu E upregulates LC3B-II and p62 expression, suggesting an incomplete autophagy in A549 cells (Figure 5A,B). Knockdown of ATG5 rescued the cells from Cu E-mediated growth inhibition and apoptosis (Figure 6A,B). Because the knockdown efficiency of the ATG5 gene is only 50% in A549 cells, we confirmed the findings by comparing the effects of Cu E on proliferation, autophagy, and apoptosis in WT-MEF and ATG5KO-MEF. The data further support our hypothesis that ATG5-knockout rescued the cells from Cu E-mediated anti-proliferative activity, incomplete autophagy, and apoptosis (Figure S3). Importantly, p62-knockdown decreased Cu E-induced ROS production, DNA damage, and apoptosis (Figure 6C–E). Therefore, this is the first study showing that Cu E promotes incomplete autophagy and that ultimately causes cell death in A549 cells. Interestingly, cucurbitacin I reportedly induces pro-death autophagy in A549 cells by inhibiting ERK/mTOR/STAT3 pathway [57]. Furthermore, Cu E has been previously observed to possess neuroprotective properties in dopaminergic neurons by inhibiting autophagic flux, as indicated by the accumulation of large vacuoles and elevated p62 levels [58]. The molecular mechanisms of incomplete autophagy include triggering the accumulation of autophagosomes, inhibiting the fusion of autophagosomes with lysosomes, and impairing the function of lysosomes [56]. The biogenesis and trafficking of autophagosomes rely on various cytoskeletal components, such as actin filaments and microtubules [59,60]. Cu E has been reported to inhibit actin filament depolymerization [61] and interfere with microtubule assembly during growth and steady state [54]. Therefore, Cu E may disrupt the process of autophagy by affecting the actin cytoskeleton and microtubules.

## 5. Conclusions

In this study, we made significant observations regarding the effects of Cu E on A549 cells. We found that Cu E effectively inhibits the viability and proliferation of A549 cells, leading to cell cycle arrest at the G2/M and subG1 phases. These effects are associated with the inhibition of phosphorylation and reduction in protein levels of regulatory proteins, thereby impeding G2/M cell-cycle progression. Additionally, we observed that Cu E-induced incomplete autophagy plays a critical role in promoting ROS production, DNA damage, and apoptosis. Based on these findings, we propose that Cu E holds promise as a potential drug candidate for the treatment of NSCLC.

## Data Availability

The data that support the findings of this study are available from the corresponding author upon reasonable request.

## Supplementary Materials

The following supporting information can be downloaded at: https://www.mdpi.com/article/10.3390/cimb45100514/s1, Figure S1: the effects of Cu E on cell proliferation in NCI-H1299, NCI-H226, NCI-1437, and BEAS-2B cells. Figure S2: time-course study of Cu E on cell-cycle progression in A549 cells. Figure S3: the effects of Cu E in wild-type MEF (MEF-WT) and ATG5-knockout MEF (MEF-ATG5KO).

## Abbreviations

ATCC, American Type Culture Collection; Cu E, Cucurbitacin E; EGFR-TKIs, EGFR tyrosine kinase inhibitors; FBS, fetal bovine serum; MEF, mouse embryonic fibroblast; MTT, 3-(4,5-Dimethylthiazol-2-yl)-2,5-diphenyltetrazolium bromide; NSCLC, non-small-cell lung cancer; SRB, Sulforhodamine B.

## References

[1] Sung H., Ferlay J., Siegel R.L., Laversanne M., Soerjomataram I., Jemal A., Bray F. (2021). Global Cancer Statistics 2020: GLOBOCAN Estimates of Incidence and Mortality Worldwide for 36 Cancers in 185 Countries. CA Cancer J. Clin..

[2] Siegel R.L., Miller K.D., Wagle N.S., Jemal A. (2023). Cancer statistics, 2023. CA Cancer J. Clin..

[3] Travis W.D. (2020). Lung Cancer Pathology: Current Concepts. Clin. Chest Med..

[4] Kashima J., Kitadai R., Okuma Y. (2019). Molecular and Morphological Profiling of Lung Cancer: A Foundation for “Next-Generation” Pathologists and Oncologists. Cancers.

[5] Tan A.C., Tan D.S.W. (2022). Targeted Therapies for Lung Cancer Patients with Oncogenic Driver Molecular Alterations. J. Clin. Oncol..

[6] Oxnard G.R., Hu Y., Mileham K.F., Husain H., Costa D.B., Tracy P., Feeney N., Sholl L.M., Dahlberg S.E., Redig A.J. (2018). Assessment of Resistance Mechanisms and Clinical Implications in Patients With EGFR T790M-Positive Lung Cancer and Acquired Resistance to Osimertinib. JAMA Oncol..

[7] Asao T., Takahashi F., Takahashi K. (2019). Resistance to molecularly targeted therapy in non-small-cell lung cancer. Respir. Investig..

[8] Yang S.H. (2013). Molecular basis of drug resistance: Epidermal growth factor receptor tyrosine kinase inhibitors and anaplastic lymphoma kinase inhibitors. Tuberc. Respir. Dis..

[9] Newman D.J., Cragg G.M. (2012). Natural products as sources of new drugs over the 30 years from 1981 to 2010. J. Nat. Prod..

[10] Newman D.J., Cragg G.M. (2020). Natural Products as Sources of New Drugs over the Nearly Four Decades from 01/1981 to 09/2019. J. Nat. Prod..

[11] Mushtaq S., Abbasi B.H., Uzair B., Abbasi R. (2018). Natural products as reservoirs of novel therapeutic agents. EXCLI J..

[12] Wen T., Song L., Hua S. (2021). Perspectives and controversies regarding the use of natural products for the treatment of lung cancer. Cancer Med..

[13] Umar A., Dunn B.K., Greenwald P. (2012). Future directions in cancer prevention. Nat. Rev. Cancer.

[14] Haddad R.I., Shin D.M. (2008). Recent advances in head and neck cancer. N. Engl. J. Med..

[15] Glade M.J. (1999). Food, nutrition, and the prevention of cancer: A global perspective. American Institute for Cancer Research/World Cancer Research Fund, American Institute for Cancer Research, 1997. Nutrition.

[16] Reddy A.S., Zhang S. (2013). Polypharmacology: Drug discovery for the future. Expert Rev. Clin. Pharmacol..

[17] Surh Y.J. (2003). Cancer chemoprevention with dietary phytochemicals. Nat. Rev. Cancer.

[18] Villanueva C., Vuillemin A.T., Demarchi M., Bazan F., Chaigneau L., Pivot X. (2009). Ixabepilone: A new active chemotherapy in the treatment of breast cancer. Women’s Health.

[19] Swami U., Chaudhary I., Ghalib M.H., Goel S. (2012). Eribulin—A review of preclinical and clinical studies. Crit. Rev. Oncol. Hematol..

[20] Chen X., Bao J., Guo J., Ding Q., Lu J., Huang M., Wang Y. (2012). Biological activities and potential molecular targets of cucurbitacins: A focus on cancer. Anticancer Drugs.

[21] Alghasham A.A. (2013). Cucurbitacins—A promising target for cancer therapy. Int. J. Health Sci..

[22] Kumar A., Sharma B., Sharma U., Parashar G., Parashar N.C., Rani I., Ramniwas S., Kaur S., Haque S., Tuli H.S. (2023). Apoptotic and antimetastatic effect of cucurbitacins in cancer: Recent trends and advancement. Naunyn-Schmiedeberg’s Arch. Pharmacol..

[23] Mohamed G.A., Ibrahim S.R.M., El-Agamy D.S., Elsaed W.M., Sirwi A., Asfour H.Z., Koshak A.E., Elhady S.S. (2022). Cucurbitacin E glucoside alleviates concanavalin A-induced hepatitis through enhancing SIRT1/Nrf2/HO-1 and inhibiting NF-kB/NLRP3 signaling pathways. J. Ethnopharmacol..

[24] Qiao J., Xu L.H., He J., Ouyang D.Y., He X.H. (2013). Cucurbitacin E exhibits anti-inflammatory effect in RAW 264.7 cells via suppression of NF-kappaB nuclear translocation. Inflamm. Res..

[25] Liu Z., Kumar M., Devi S., Kabra A. (2021). The Mechanisms of Cucurbitacin E as a Neuroprotective and Memory-Enhancing Agent in a Cerebral Hypoperfusion Rat Model: Attenuation of Oxidative Stress, Inflammation, and Excitotoxicity. Front. Pharmacol..

[26] Varela C., Melim C., Neves B.G., Sharifi-Rad J., Calina D., Mamurova A., Cabral C. (2022). Cucurbitacins as potential anticancer agents: New insights on molecular mechanisms. J. Transl. Med..

[27] Huang W.W., Yang J.S., Lin M.W., Chen P.Y., Chiou S.M., Chueh F.S., Lan Y.H., Pai S.J., Tsuzuki M., Ho W.J. (2012). Cucurbitacin E Induces G(2)/M Phase Arrest through STAT3/p53/p21 Signaling and Provokes Apoptosis via Fas/CD95 and Mitochondria-Dependent Pathways in Human Bladder Cancer T24 Cells. Evid. Based Complement. Altern. Med..

[28] Liu Y., Yang H., Guo Q., Liu T., Jiang Y., Zhao M., Zeng K., Tu P. (2020). Cucurbitacin E Inhibits Huh7 Hepatoma Carcinoma Cell Proliferation and Metastasis via Suppressing MAPKs and JAK/STAT3 Pathways. Molecules.

[29] Zhang L., Liang H., Xin Y. (2020). Cucurbitacin E inhibits esophageal carcinoma cell proliferation, migration, and invasion by suppressing Rac1 expression through PI3K/AKT/mTOR pathway. Anticancer Drugs.

[30] Yang P., Lian Q., Fu R., Ding G.B., Amin S., Li Z., Li Z. (2022). Cucurbitacin E Triggers Cellular Senescence in Colon Cancer Cells via Regulating the miR-371b-5p/TFAP4 Signaling Pathway. J. Agric. Food Chem..

[31] Zha Q.B., Zhang X.Y., Lin Q.R., Xu L.H., Zhao G.X., Pan H., Zhou D., Ouyang D.Y., Liu Z.H., He X.H. (2015). Cucurbitacin E Induces Autophagy via Downregulating mTORC1 Signaling and Upregulating AMPK Activity. PLoS ONE.

[32] Dong Y., Lu B., Zhang X., Zhang J., Lai L., Li D., Wu Y., Song Y., Luo J., Pang X. (2010). Cucurbitacin E, a tetracyclic triterpenes compound from Chinese medicine, inhibits tumor angiogenesis through VEGFR2-mediated Jak2-STAT3 signaling pathway. Carcinogenesis.

[33] Zhang T., Li J., Dong Y., Zhai D., Lai L., Dai F., Deng H., Chen Y., Liu M., Yi Z. (2012). Cucurbitacin E inhibits breast tumor metastasis by suppressing cell migration and invasion. Breast Cancer Res. Treat..

[34] Chun Y., Kim J. (2018). Autophagy: An Essential Degradation Program for Cellular Homeostasis and Life. Cells.

[35] Noda N.N., Inagaki F. (2015). Mechanisms of Autophagy. Annu. Rev. Biophys..

[36] Dikic I., Elazar Z. (2018). Mechanism and medical implications of mammalian autophagy. Nat. Rev. Mol. Cell Biol..

[37] Galluzzi L., Bravo-San Pedro J.M., Levine B., Green D.R., Kroemer G. (2017). Pharmacological modulation of autophagy: Therapeutic potential and persisting obstacles. Nat. Rev. Drug Discov..

[38] Song H., Sui H., Zhang Q., Wang P., Wang F. (2020). Cucurbitacin E Induces Autophagy-Involved Apoptosis in Intestinal Epithelial Cells. Front. Physiol..

[39] Ma G., Luo W., Lu J., Ma D.L., Leung C.H., Wang Y., Chen X. (2016). Cucurbitacin E induces caspase-dependent apoptosis and protective autophagy mediated by ROS in lung cancer cells. Chem. Biol. Interact..

[40] Zhang J., Aray B., Zhang Y., Bai Y., Yuan T., Ding S., Xue Y., Huang X., Li Z. (2023). Synergistic effect of cucurbitacin E and myricetin on Anti-Non-Small cell lung cancer: Molecular mechanism and therapeutic potential. Phytomedicine.

[41] Chang C.H., Lin B.J., Chen C.H., Nguyen N.L., Hsieh T.H., Su J.H., Chen M.C. (2023). Stellettin B Induces Cell Death in Bladder Cancer Via Activating the Autophagy/DAPK2/Apoptosis Signaling Cascade. Mar. Drugs.

[42] Chen M.C., Lin Y.C., Liao Y.H., Liou J.P., Chen C.H. (2019). MPT0G612, a Novel HDAC6 Inhibitor, Induces Apoptosis and Suppresses IFN-gamma-Induced Programmed Death-Ligand 1 in Human Colorectal Carcinoma Cells. Cancers.

[43] Li J., Pan Y.Y., Zhang Y. (2013). Synergistic interaction between sorafenib and gemcitabine in EGFR-TKI-sensitive and EGFR-TKI-resistant human lung cancer cell lines. Oncol. Lett..

[44] Castedo M., Perfettini J.L., Roumier T., Kroemer G. (2002). Cyclin-dependent kinase-1: Linking apoptosis to cell cycle and mitotic catastrophe. Cell Death Differ..

[45] Joukov V., De Nicolo A. (2018). Aurora-PLK1 cascades as key signaling modules in the regulation of mitosis. Sci. Signal..

[46] Klionsky D.J., Abdel-Aziz A.K., Abdelfatah S., Abdellatif M., Abdoli A., Abel S., Abeliovich H., Abildgaard M.H., Abudu Y.P., Acevedo-Arozena A. (2021). Guidelines for the use and interpretation of assays for monitoring autophagy (4th edition)^1^. Autophagy.

[47] Chen C.H., Liu Y.M., Pan S.L., Liu Y.R., Liou J.P., Yen Y. (2016). Trichlorobenzene-substituted azaaryl compounds as novel FGFR inhibitors exhibiting potent antitumor activity in bladder cancer cells in vitro and in vivo. Oncotarget.

[48] Kongara S., Karantza V. (2012). The interplay between autophagy and ROS in tumorigenesis. Front. Oncol..

[49] Mathew R., Karp C.M., Beaudoin B., Vuong N., Chen G., Chen H.Y., Bray K., Reddy A., Bhanot G., Gelinas C. (2009). Autophagy suppresses tumorigenesis through elimination of p62. Cell.

[50] Chen C.H., Changou C.A., Hsieh T.H., Lee Y.C., Chu C.Y., Hsu K.C., Wang H.C., Lin Y.C., Lo Y.N., Liu Y.R. (2018). Dual Inhibition of PIK3C3 and FGFR as a New Therapeutic Approach to Treat Bladder Cancer. Clin. Cancer Res..

[51] Yang Y., Zhang H., Huang S., Chu Q. (2023). KRAS Mutations in Solid Tumors: Characteristics, Current Therapeutic Strategy, and Potential Treatment Exploration. J. Clin. Med..

[52] Kong Y., Chen J., Zhou Z., Xia H., Qiu M.H., Chen C. (2014). Cucurbitacin E induces cell cycle G2/M phase arrest and apoptosis in triple negative breast cancer. PLoS ONE.

[53] Cheng A.C., Hsu Y.C., Tsai C.C. (2019). The effects of cucurbitacin E on GADD45beta-trigger G2/M arrest and JNK-independent pathway in brain cancer cells. J. Cell. Mol. Med..

[54] Wang X., Tanaka M., Peixoto H.S., Wink M. (2017). Cucurbitacins: Elucidation of their interactions with the cytoskeleton. PeerJ.

[55] Dey P. (2004). Aneuploidy and malignancy: An unsolved equation. J. Clin. Pathol..

[56] Zhang Q., Cao S., Qiu F., Kang N. (2022). Incomplete autophagy: Trouble is a friend. Med. Res. Rev..

[57] Ni Y., Wu S., Wang X., Zhu G., Chen X., Ding Y., Jiang W. (2018). Cucurbitacin I induces pro-death autophagy in A549 cells via the ERK-mTOR-STAT3 signaling pathway. J. Cell. Biochem..

[58] Arel-Dubeau A.M., Longpre F., Bournival J., Tremblay C., Demers-Lamarche J., Haskova P., Attard E., Germain M., Martinoli M.G. (2014). Cucurbitacin E has neuroprotective properties and autophagic modulating activities on dopaminergic neurons. Oxidative Med. Cell. Longev..

[59] Kast D.J., Dominguez R. (2017). The Cytoskeleton-Autophagy Connection. Curr. Biol..

[60] Trisciuoglio D., Degrassi F. (2021). The Tubulin Code and Tubulin-Modifying Enzymes in Autophagy and Cancer. Cancers.

[61] Sorensen P.M., Iacob R.E., Fritzsche M., Engen J.R., Brieher W.M., Charras G., Eggert U.S. (2012). The natural product cucurbitacin E inhibits depolymerization of actin filaments. ACS Chem. Biol..

