# Supplementary material for: Cucurbitacin E Exerts Anti-Proliferative Activity via Promoting p62-Dependent Apoptosis in Human Non-Small-Cell Lung Cancer A549 Cells
Source: Curr Issues Mol Biol. 2023 Oct 7;45(10):8138–51. doi: 10.3390/cimb45100514 (PMC10605876; doi:10.3390/cimb45100514)
Supplement: Supplementary file 1 [file cimb-45-00514-s001.zip › cimb-2656934-SI.pdf]

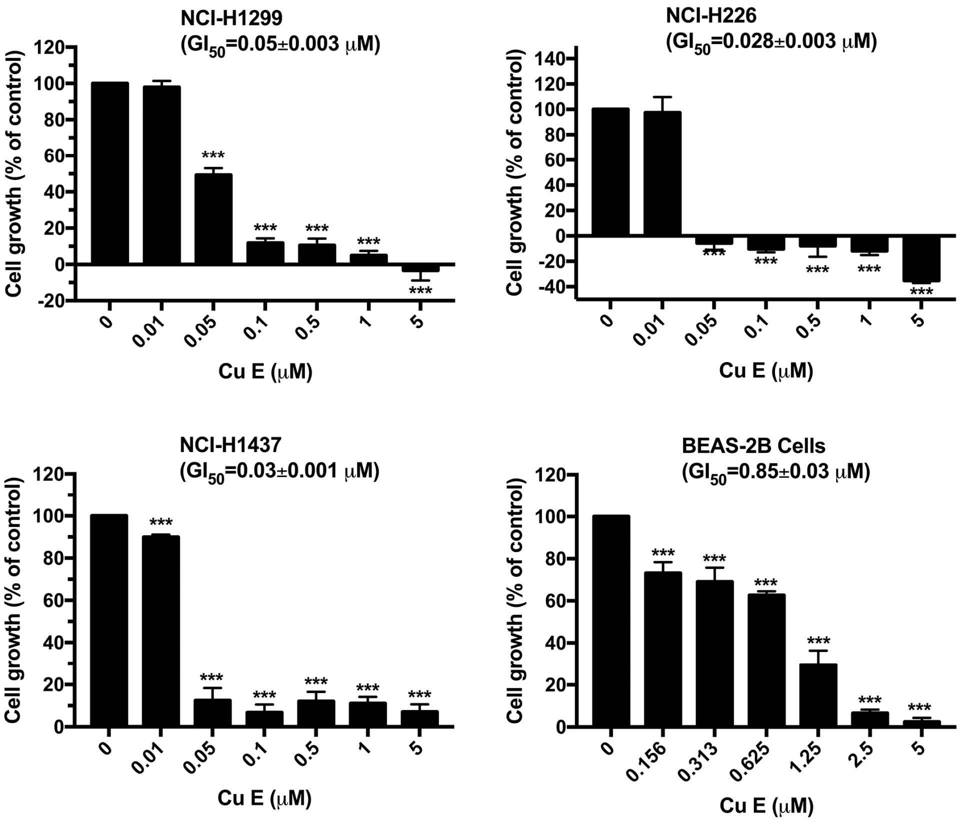

Figure S1. The effects of Cu E on cell proliferation in NCI-H1299, NCI-H226, NCI-1437, and BEAS-2B cells. The cells were treated with different concentrations of Cu E for 48 h, and cell growth was determined through SRB assay. The data are presented as the mean ± S.D. (n=3) *** $p < 0.001$ compared with control group.

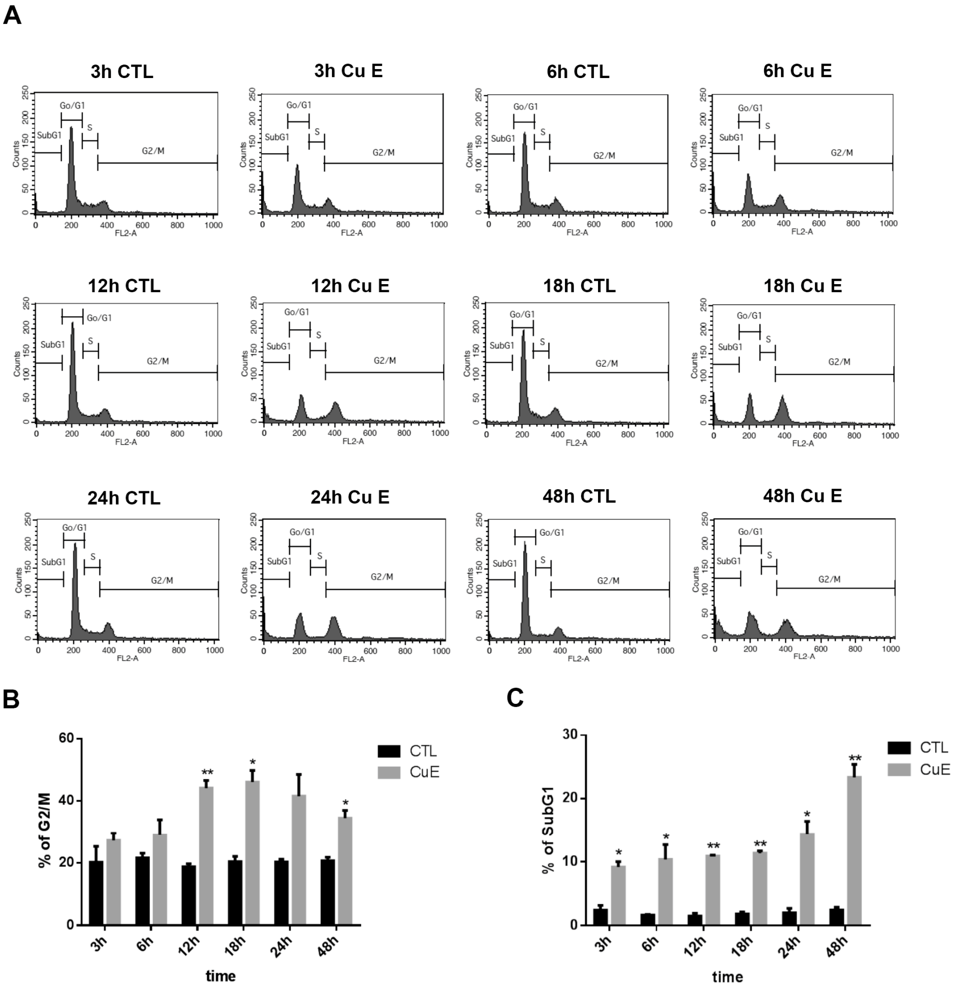

Figure S2. Time-course study of Cu E on cell-cycle progression in A549 cells. **(A)** The cells were exposed to the 1 μM of Cu E for the indicated time periods, and cell-cycle distribution was analyzed using flow cytometry after propidium iodide (PI) staining. The data showed that Cu E promotes cell-cycle arrest at G2/M phase **(B)** and subG1 phase **(C)**. The data are presented as the mean ± S.D. (n=2) * $p < 0.05$, and ** $p < 0.01$ compared with control group.

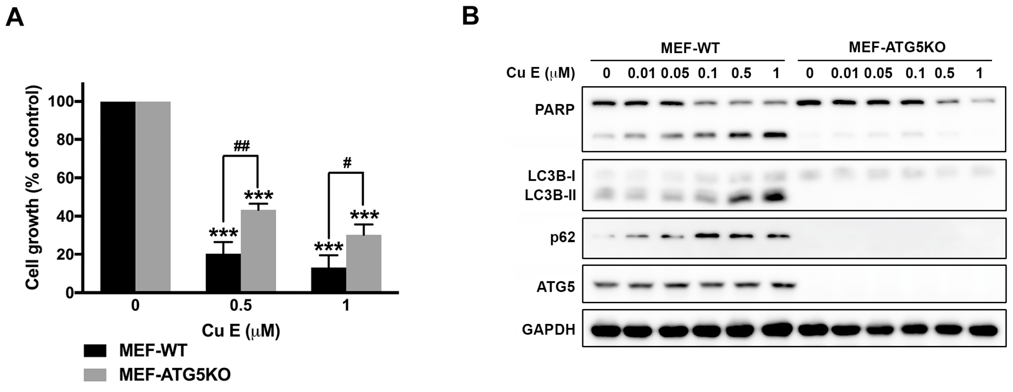

Figure S3. The effects of Cu E in wild-type MEF (MEF-WT) and ATG5-knockout MEF (MEF-ATG5KO). **(A)** The cells were exposed to indicated concentrations of Cu E for 24 h, and cell growth was examined by SRB assay. The data are expressed as means ± S.D. (n=3) ***, $p < 0.001$ compared to control group; #, $p < 0.05$; ##, $p < 0.01$ compared to MEF-WT cells. (B) The cells were exposed to indicated concentrations of Cu E for 24 h, and cell lysates were immunoblotted using the indicated antibodies.
